# A Participatory Regional Partnership Approach to Promote Nutrition and Physical Activity Through Environmental and Policy Change in Rural Missouri

**DOI:** 10.5888/pcd12.140593

**Published:** 2015-06-11

**Authors:** Ellen K. Barnidge, Elizabeth A. Baker, Amy Estlund, Freda Motton, Pamela R. Hipp, Ross C. Brownson

**Affiliations:** Author Affiliations: Elizabeth A. Baker, Amy Estlund, Freda Motton, Prevention Research Center in St Louis, Saint Louis University College for Public Health and Social Justice, St Louis, Missouri; Pamela R. Hipp, Prevention Research Center in St Louis, Brown School, Washington University in St Louis, St. Louis, Missouri; Ross C. Brownson, Prevention Research Center in St Louis, Brown School, Washington University in St Louis, and Division of Public Health Sciences and Alvin J. Siteman Cancer Center, Washington University School of Medicine, Washington University in St. Louis, St. Louis, Missouri.

## Abstract

**Background:**

Rural residents are less likely than urban and suburban residents to meet recommendations for nutrition and physical activity. Interventions at the environmental and policy level create environments that support healthy eating and physical activity.

**Community Context:**

Healthier Missouri Communities (Healthier MO) is a community-based research project conducted by the Prevention Research Center in St. Louis with community partners from 12 counties in rural southeast Missouri. We created a regional partnership to leverage resources and enhance environmental and policy interventions to improve nutrition and physical activity in rural southeast Missouri.

**Methods:**

Partners were engaged in a participatory action planning process that included prioritizing, implementing, and evaluating promising evidence-based interventions to promote nutrition and physical activity. Group interviews were conducted with Healthier MO community partners post intervention to evaluate resource sharing and sustainability efforts of the regional partnership.

**Outcome:**

Community partners identified the benefits and challenges of resource sharing within the regional partnership as well as the opportunities and threats to long-term partnership sustainability. The partners noted that the regional participatory process was difficult, but the benefits outweighed the challenges.

**Interpretation:**

Regional rural partnerships may be an effective way to leverage relationships to increase the capacity of rural communities to implement environmental and policy interventions to promote nutrition and physical activity.

## Background

Chronic diseases (eg, heart disease, cancer, stroke) account for most premature deaths (1). Although research demonstrates the association of risky behavior with chronic disease, particularly physical inactivity and poor nutrition, changing these behaviors is challenging, particularly in rural communities where residents are less likely to meet recommendations for these behavioral determinants (2–4).

Failure to meet the recommendations is due to factors across several ecological levels. At an individual level, rural residents have limited exposure to preventive health care messages (5). Rural residents also have limited access to environmental determinants of physical activity or healthy eating, such as safe, walkable communities, recreation facilities (including informal recreation areas such as hiking trails), parks, and healthful food outlets (5–7). Creating environmental supports and changing policy in rural communities is particularly challenging because rural communities have lower population density and, thus, fewer resources than their urban and suburban counterparts. Our case study describes how we created a regional partnership to leverage resources and enhance environmental and policy initiatives to improve nutrition and physical activity in rural Missouri.

## Community Context

**Figure 1:**
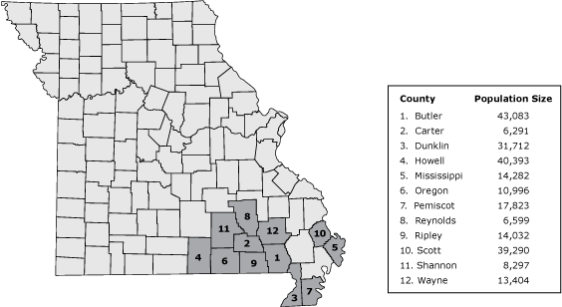
Counties in the Healthier Missouri Communities partnership and US Census Bureau population estimates for each county (15). **Table:** 

Healthier Missouri Communities (Healthier MO) is a community-based research project conducted by the Prevention Research Center in St. Louis (PRC-StL) and community partners from 12 counties in rural southeast Missouri (Figure 1). Healthier MO was supported by funding from the Centers for Disease Control and Prevention through the Prevention Research Center Office. The PRC-StL began working with community partners in southeast Missouri in 1994 through county heart health coalitions. The PRC-StL built partnerships in southeast Missouri because of the high poverty rate (currently double the Missouri rate of 15%) (8) and significantly higher rates of chronic disease than the rest of the state (9). All counties are rural (10) and currently all but one are designated as Medically Underserved Areas (11). Southeast Missouri residents are less likely to be physically active than Missouri residents as a whole (12) and, although county data for fruit and vegetable consumption is unavailable, Missouri residents overall are less likely to meet recommendations for fruit and vegetable consumption than residents in the nation as a whole (13). Access to places to be physically active (eg, parks and recreational facilities) varies across the region, and residents have less access to healthful food than do residents in other regions in the state (14).

County, Each Located in Southeastern Missouri	Population Size
Butler	43,083
Carter	6,291
Dunklin	31,712
Howell	40,393
Mississippi	14,282
Oregon	10,996
Pemiscot	17,823
Reynolds	6,599
Ripley	14,032
Scott	39,290
Shannon	8,297
Wayne	13,404

This article describes the partnership activities from 2010 to 2014. Before 2010, four of the initial heart health coalitions pilot-tested a regional partnership approach. For the 2010–2014 funding cycle, a decision to shift to a regional partnership approach with all counties was made by community partners and informed by interviews conducted with national key informants who were implementing environmental or policy change to promote nutrition and physical activity (16). The interview findings suggested that rural communities face a lack of human capital, professional training opportunities, and the perception that policy changes have a small impact on population health because rural populations are small (compared with suburban and urban populations). Informants recommended developing a broad-based partnership to leverage regional resources and increase the health impact of environmental or policy changes (16). The collective decision to move toward a regional partnership also marked a transition from community-based research to community-based participatory research (CBPR).

The programmatic objective of Healthier MO was to implement environmental and policy interventions to promote physical activity and healthy eating across southeast Missouri. The partnership engagement objective was to develop a regional partnership with representation from the 12 counties to design, implement, and evaluate interventions to promote these behaviors. The anticipated outcomes of the partnership engagement component were 1) increased resource sharing among partners and 2) a sustainable regional partnership focusing on chronic disease prevention.

## Methods

### Evidence-based training

**Figure 2:**
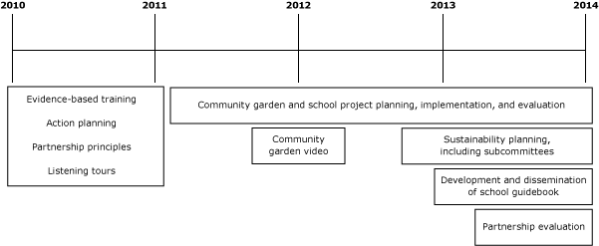
Timeline of Healthier Missouri Communities partnership activities from 2010 to 2014.

In spring 2010, thirty community partners representing the 12 Healthier MO counties participated in an evidence-based decision-making training, using a well-established training model based on *Evidence-Based Public Health* (Figure 2) (17). Community partners included those who participated in the county heart health coalitions before 2010 and others doing similar work in the region. The partnership included African American and white partners representing grassroots communities, community-based organizations, schools, and health departments.

The training focused on essential elements of evidence-based public health and advantages of adapting evidence-based programs for one’s community. Academic partners engaged community partners in an evidence-based decision-making process to determine which environmental or policy interventions addressing nutrition and physical activity were feasible and important for their region. The community partners identified community gardens and school wellness initiatives. Partners also learned about CBPR and the process of using the approach.

### Partnership engagement

The partners previously engaged in the county heart health coalitions recognized CBPR as a significant shift. For the first time community partners engaged in the design, implementation, and evaluation of the intervention, a common practice in a CBPR approach. Also, there was a shift in the way counties were compensated. Previously counties were provided with funding to engage in academically defined interventions and given small stipends to spend on nutrition and physical activity activities of their choosing. In Healthier MO, partners collectively decided on acceptable expenditures based on regional efforts with community gardens and school wellness initiatives.

The Healthier MO partnership was staffed by a research team that included academic staff and faculty and 2 community liaisons. All meeting times, locations, and topics, were determined by all partners, with the research team developing meeting agendas and managing meeting logistics. Communication was facilitated by the research team, including distribution of notes and documentation of decisions made by the partnership. Individual community partners disseminated information to their community constituents. A team of research and community partners worked with local media to disseminate information about events and successes with a larger regional audience.

### Action planning and implementation

Partners met monthly after community gardens and school wellness were identified as the intervention foci. During meetings in fall 2010 and spring 2011, the partners developed partnership principles to determine how they would work together (Table) and an action plan to define the objectives; action steps to design, implement, and evaluate the chosen interventions; and the outcomes of interest. In summer 2011, the research staff conducted listening tours in each community to evaluate the action planning process and understand each community’s hopes for the regional partnership. The listening tours solidified participation in the partnership and buy-in from local community members who were not representing their communities at the monthly meetings. During the action planning phase, the composition of the partnership changed. Whereas some of the partners who participated in the heart health coalitions determined the process or purpose no longer fit their local community’s needs, new members joined upon invitation by community partners who remained committed. The partners decided to engage new partners as appropriate without extensive recruitment.

**Table:** Partnership Principles Developed by Healthier Missouri Community Partners, 2010–2014

Principle	Operationalization
**Develop and maintain trust**	Be accountable (say what you’re going to do, and do what you say).
	Be honest, open, and respectful. If you state something as a fact, be sure it is a fact.
	Stick to the agenda.
	Make sure clear communication is established — be careful with emails because things can get lost or misinterpreted in emails.
	Get feedback from other perspectives.
	Assume good intentions of all partners and do not assume negatives.
**Provide shared leadership**	**As a facilitator**
	Guide the group, help pull out ideas, and keep the group focused.
	Be passionate about the project.
	**As a member**
	Give ideas.
	Be passionate about the project.
	Know the strengths of each member, and play off those strengths.
	Foster long-term relationships.
**Develop processes for shared power and influence**	Empower everyone to speak and share their experiences with the group — face-to-face is best.
	Make everybody feel welcome and welcome all perspectives, although they may be different.
	Allow different people to represent a community — maybe a co-chair from a heart health coalition or a community garden representative.
	Inform, welcome, and include new people in the process when they rotate into meetings.
	Take and distribute notes.
	Write down vision and mission.
**Address conflict**	Meet face-to-face to help explain and clarify and reduce conflict.
	Identify the issues and solve them maturely and as adults.
	Create an environment that allows participants to agree and disagree and work on the fact that what works in one county may not work for another.
	Do not get caught up in the problem, but focus on solutions instead.
	Create a place to voice concerns and feel comfortable without worrying about being shot down or made to feel stupid.
	Agree to a certain amount of time to address issues and then agree to move on.
	Create an open sharing time on the agenda to address concerns.
	Agree to disagree.
	Be respectful.
**Establish shared decision making processes**	Agree that people who are present at meeting will vote.
	Send information before meetings about big decisions being discussed or made at the meeting to ensure people are aware of what may come up for a vote.
	Use thumbs up, thumbs down, or thumbs sideways approach to make group decisions. Thumbs sideways means more information is needed.
	Use majority rule, but realize that things are flexible if the group wants to change majority rule on an issue.
	Agree that there are some issues where everyone needs to agree.
	Use emails, faxes, postal mail, texts, or telephone calls to inform group members, and allow time to ask questions and discuss before decisions are made.
**Evaluation of process, impact, and outcome**	Agree to look at the end goal first and constantly review.
	Commit to thinking about process too. Consider how the team is working as a group as well as what the team is doing.
	Plan internally for staff changes to ensure intended outcomes are reached.

From 2011 to 2014, the partners implemented and evaluated a series of intervention activities. The monthly meetings were used to support the implementation, share information, provide training, and coordinate evaluation. Those involved in community gardens tracked planting and harvest data, and teachers implementing the school intervention tracked student participation and, in some cases, outcome data. At least once per year academic partners analyzed data and formally presented findings to the community partners, giving community partners an opportunity to discuss them. In 2012, partners used community garden evaluation data to develop a video as a marketing and dissemination tool.

In 2013, garden and school subcommittees were formed to focus on dissemination of the research findings and to plan for partnership sustainability when grant funding concluded. Healthier MO members volunteered to join the subcommittees and get involved in the work between partnership meetings. The subcommittees discussed ongoing funding and partnership structure and process. The school subcommittee developed a guidebook to disseminate the results of the school wellness interventions to increase physical activity during the school day.

### Assessment of partnership engagement

In spring 2014 two group interviews were conducted with Healthier MO community partners. Of the 20 community partners, 12 participated in the group interviews, and 3 partners participated separately by telephone or electronic survey. The purpose of the group interviews was to assess the benefits and challenges of the regional partnership approach and the perceived outcomes of the partnership. Partnership literature (18,19) informed the development of the interview guide, and focused coding was used to code data. The research protocol was approved by the academic partners’ institutional review boards.

## Outcome

### Resource sharing

The partners discussed benefits and challenges of sharing resources in the form of social support, information, expertise, and skills as part of a participatory regional partnership. Healthier MO partners traveled up to 2½ hours to attend monthly meetings. Some participants noted that the benefit of the regional partnership was evidenced by the fact that people showed up each month. As one member stated, “This is just a very small part of my organization, but the benefits of coming far, far, far outweigh the challenge of finding the time.” One participant highlighted the social support received by bringing people together regionally. The partner shared that the regional partnership is energizing and helps when managing small struggling local efforts. Participants also noted that the partnership benefited them personally by helping to develop their professional networks, for example, with schools. Overall, partners expressed that time provided at meetings to communicate one-on-one created space “where we shared our struggles and our triumphs.”

Other participants noted the benefit of sharing information and expertise. One participant described the benefit as “being able to share best practices as far as what other groups are doing.” A local health department partner found it particularly beneficial to learn what other health departments were doing. Several partners noted it was not just about one-way information sharing but “we threw ideas or problems out and someone would say, ‘This is what I did for that.’” Another partner said that information helped “my direction, opened up ideas that I wouldn’t have come up with, gave me encouragement and it gave us confidence to go forward and have some kind of feedback from people who might be doing it differently or better.” In addition to informal information sharing, partners indicated the benefit of formal trainings on such topics as grant writing. The partners noted that because of information sharing in the meetings they called one another in between meetings for advice. One partner emphasized that the information and support received by the regional partners was a greater benefit than the grant funds.

The shift to a regional approach also presented challenges. Partners explained that the original county heart health coalitions got money and were not required to participate in the work of the partnership. One participant explained, “I think a lot of people were still in the mindset of the heart health coalition where they just got some money and then they could go do what they wanted with it, and they didn’t have to really participate.” A few partners also indicated that being rural made it harder than initially thought to share resources and information because of the geographical distance between partners.

### Sustainability

The second outcome was the sustainability of the Healthier MO partnership after the funding ended. The partnership prepared by developing subcommittees for each initiative (community gardens and schools) and developing a leadership structure for the next partnership incarnation. In the post group interviews, participants identified future areas of consideration, including funding, partnership structure and function, and relationship building.

Partners expressed that lack of funding would be a challenge to long-term partnership sustainability. Partners were concerned about how people will travel without mileage reimbursement. Another member explained that some partners are able to participate because of the funding and may not be able to justify the time to their organization without funding. The partners did identify ways to generate funds. One participant suggested partners can sell fruits and vegetables from the community gardens to sustain the partnership when grant funds end. A second participant noted that state funders may be more interested in funding Healthier MO because it is a regional initiative. However, members noted that the partnership will need to enhance their capacity to submit and manage grants.

Partnership structure and function were identified as main sustainability concerns. Partners were concerned about long-term facilitation and organization. Several partners shared that although they can participate, they cannot lead due to other organizational commitments. One partner suggested that a paid leader might be needed while several others suggested a group of partners is needed to lead. One participant acknowledged that delegating roles will be important, stating, “I do think it would be a good idea to have someone in charge of communicating, someone in charge of finances, and in charge of the meetings themselves, getting the agendas.” The partners determined that moving forward they will meet quarterly. As a result, one partner explained the meetings will have to produce results. She noted, “we can’t say, ‘oh, we’ll table that to next month,’ because then it’ll be too late.” Another noted that people need to show up to meetings, stating, “I think it’s very important that because we’re not having as many meetings that when we do have them, we need to come together, because if we don’t, that’s how it’s going to fall apart.” To come together and communicate between meetings, partners identified text messaging as a “plus” because some members do not have Internet access. Despite the challenges, partners expressed that they have “finally bonded” and they will participate, regardless of funding.

### Intervention outcomes of a regional rural partnership

The participants identified outcomes of the interventions, including those related to community gardens and schools. Partners noted that existing community gardens improved and the region gained new gardens. Another participant noted that the gardens allowed community members “to live a healthier lifestyle . . . eating healthier, growing their own produce” and provided access to fresh fruits and vegetables to folks that would not have access. Another mentioned that the community gardens increased physical activity for older adults in their community. A participant noted that the school initiative was important for children to learn physical activity at an early age to build the skills that they can maintain in the future. Another partner noted that the playground equipment and the walking tracks installed at 4 schools will have long-term benefits.

## Interpretation

Healthier MO was a shift to a participatory regional partnership approach that intended to build the southeast Missouri regional capacity to promote physical activity and healthy eating through environmental and policy change. Partnership outcomes were increased resource sharing and partnership sustainability. Although there was change in how funds were distributed and used for interventions, the funding level did not increase. However, the partners did see an increase in intangible resources, including shared expertise, knowledge, and skills from Healthier MO beyond what was available in their local rural community. The acknowledged benefit of a regional approach and the relationships and structures developed were seen as supporting short-term sustainability. The absence of funding for future travel was identified as a challenge that could reduce long-term sustainability.

Most partners agreed that being highly engaged in the design, implementation, and evaluation increased a sense of ownership among all partners. Perhaps as a result of this sense of ownership, community partners noted a missed opportunity to plan ahead. Although Healthier MO initiated planning for partnership sustainability approximately 2 years before the formal funding end, partners agreed that planning should have started earlier. For example, one community partner suggested that a broader community–academic facilitation model be implemented initially. Because of the dramatic shift in community engagement at Healthier MO’s inception, the research team facilitated the partnership to reduce the burden on community partners during the transition to a new participatory and regional norm. The research team included 2 paid community liaisons who were regional residents. Distributing paid positions among academic and community partners facilitates a shared benefit, and paid staff is helpful to fledgling partnerships. One drawback of paid leadership is that it can limit nonpaid partner involvement and ownership in the long term (19). The community partners noted this tension when they discussed future leadership structure.

Community partners identified 2 lessons learned that may benefit other rural areas considering a regional partnership approach. First, community members identified the need to invite funders to the partnership to help develop regional capacity to successfully apply and manage grants. In an earlier study, national rural key informants observed that smaller population size was a barrier to attracting funders because the effect on a small rural population has only a small influence on state or federal policy (16). However, eliminating rural health disparities is a national priority, and rural communities recognize that funders are necessary partners in addressing rural health gaps (20). Second, partners noted that it is essential to commit meeting time to building relationships. One partner described it as “sealing the bonds . . . so that once the funding is gone, the bonds will hold and we’ll still push forward.” This advice is supported by literature on social capital that finds that communities with higher levels of social capital or relationships of trust and reciprocity have better health outcomes (21). Relationship has long been a hallmark of small communities. Recognizing relationship building as a key capacity and leveraging it to build regional networks may be an important lesson for other rural regions in the United States.

Healthier MO demonstrates that a participatory regional partnership approach to implementing environmental and policy interventions to promote nutrition and physical activity can be successful in rural areas. A CBPR approach allowed us to increase Healthier MO partner ownership and enabled partners to choose the interventions that were most important and feasible for the region. A participatory approach also allowed us to identify what partners valued (eg, interventions, relationships, sustainability). We recommend that other rural regional partnerships discuss how partners want to be engaged and what they value early in the partnership process. In the end, Healthier MO is successful because partners value the health of the people in their region and are committed to participate regardless of the required travel, time, or funding level.
